# Long Range Correlation in Redox Potential Fluctuations Signals Energetic Efficiency of Bacterial Fe(II) Oxidation

**DOI:** 10.1038/s41598-019-40499-5

**Published:** 2019-03-11

**Authors:** Allison M. L. Enright, Brock A. Edwards, F. Grant Ferris

**Affiliations:** 1 Department of Earth and Environmental Sciences, Rutgers University - Newark, 101 Warren St., Newark, NJ 07102 USA; 2 Department of Earth Sciences, University of Toronto, 22 Russell St., Toronto, ON M5S 3B1 Canada

## Abstract

Differentiating biotic and abiotic processes in nature remains a persistent challenge, specifically in evaluating microbial contributions to geochemical processes through time. Building on previous work reporting that biologically-influenced systems exhibit stronger long-range correlation than abiotic systems, this study evaluated the relationship between long-range correlation of redox potential and oxidation rates of circumneutral microaerophilic bacterial Fe(II) oxidation using a series of batch microcosms with bacteriogenic iron oxides (BIOS). Initial detrended fluctuation analysis (DFA) scaling exponents of the abiotic microcosms were lower (ca. 1.20) than those of the biotic microcosms (ca. 1.80). As Fe(II) oxidation proceeded, correlation strength decayed as a logistic function of elapsed reaction time, exhibiting direct dependence on the free energy of reaction. Correlation strength for all microcosms decayed sharply from strong correlation to uncorrelated fluctuations. The decay rates are greater for abiotic microcosms than biotic microcosms. The Δ*G*_*m*_ relaxation edges for biotic microcosms were lower, indicating less remaining free energy for Fe(II) oxidation than abiotic systems, with the implication that biologically-catalyzed reactions are likely more energetically efficient than abiotic reactions. These results strengthen the case for employing novel DFA techniques to distinguish *in situ* microbial metabolic activity from abiotic processes, as well as to potentially differentiate metabolisms among different chemoautotrophs.

## Introduction

Deciphering the complex interplay between microbially mediated and abiotic reactions in the biogeochemical cycling of redox active chemical species is crucial to understanding the planetary impact of microbial life^1–3^. Among the multitude of redox reactions catalyzed by microorganisms, bacterial oxidation of Fe(II) is of considerable interest as Fe(II)/Fe(III) redox transformations are strongly coupled to the cycling of C, N, O, and S, as well as the environmental fate of trace elements, nutrients, and contaminants^4–6^. There is also a growing body of evidence implicating Fe(II) oxidizing bacteria (FeOB) as both causative agents in the formation of ancient banded iron formations on Earth and plausible ecophysiological analogues for life on other planets^7–10^. At the same time, the potential significance of these findings is tempered by a cautious appreciation of the fact that Fe(II) oxidation pathways in nature are exceptionally diverse and multifaceted, as concurrent abiotic and microbially-mediated reactions are apt to proceed in tandem with microaerobic, photochemical, and nitrate-dependent oxidation processes^11,12^. This reactivity extends additionally to nanoscale heterogenous oxidation processes occurring on bacterial cell and mineral surfaces^13^, further complicating these systems. Overall, distinguishing between biotic and abiotic processes *in situ* remains a persistent challenge in evaluating microbial contributions to biogeochemical processes^10^.

The high standard potential of the O_2_/H_2_O half-cell (*Eh*^0^ = 1.23V, 25 °C) promotes high rates of spontaneous abiotic oxidation of Fe(II) at atmospheric *p*O_2_ values and circumneutral pH range of most natural waters^12,14^. This constrains neutrophilic FeOB to low *p*O_2_ microaerophilic environments where the metabolic oxidation of Fe(II) can outcompete the kinetics of homogenous and heterogenous abiotic reactions^15,16^. Subsequent chemical hydrolysis and precipitation of dissolved Fe(III) often gives rise to voluminous mats of flocculent rust-coloured bacteriogenic iron oxides (BIOS) consisting of nanoparticulate hydrous ferric oxides (HFO) intermixed with live and dead bacterial cells^17–19^. The mineral precipitates occur as poorly ordered ferrihydrite^20,21^. Over time, the accumulation of BIOS serves to increase the relative proportion of heterogeneous surface-mediated abiotic reactions contributing to the abiotic oxidation of Fe(II), thereby confounding the contributions of FeOB^11^.

The kinetics of Fe(II) oxidation by neutrophilic bacteria have been examined in a number of studies^14,22–24^. Inhibition of FeOB using sodium azide as a poison or sterilization by autoclaving clearly demonstrate that rates of bacterial Fe(II) oxidation are faster than abiotic homogenous and heterogenous rates under similar geochemical and low *p*O_2_ conditions. While informative, these results only hint at the underlying mechanisms and bioenergetic capacity of FeOB to catalyze otherwise spontaneous abiotic Fe(II) oxidation rates. More recently, the statistical dependence (i.e., long-range correlation, also known as memory or persistence) of temporal fluctuations in electrochemical measurements of redox potential on a time scale of minutes to seconds were found to distinguish bacterial and abiotic Fe(II) oxidation^7,25^. Long-range correlation in a time series implies that any measured value is statistically dependent on preceding values, and can take the form of positive correlation (a past increasing trend will continue to increase into the future) or negative correlation (an increasing trend is likely to be followed by a decreasing trend)^26,27^. Such correlative dependence has proven to be consistently more pronounced for bacterial Fe(II) oxidation than abiotic Fe(II) oxidation^25^; thus, the parameter of correlation strength, measured by detrended fluctuation analysis (DFA) scaling exponents (*α*), can feasibly be used to distinguish microbially-catalyzed Fe(II) oxidation from homogenous and heterogeneous chemical oxidation. This new approach to investigating bacterial Fe(II) oxidation is compelling in light of the fact that the Gibbs free energy (Δ*G*_*r*_) of an oxidation-reduction reaction is an explicit thermodynamic function of redox potential. The implication is that Δ*G*_*r*_ reveals not only which reactions are favored thermodynamically and exist as realistic bioenergetic options, but also constrains essential geochemical conditions, including pH, ionic strength, and concentrations of electron donors and acceptors, that influence the amount of available metabolic energy.

To investigate the relationship between (Δ*G*_*r*_) and correlation strength, we conducted a series of batch microcosm experiments in which Fe(II) oxidation rates and relative concentrations of Fe(II)/Fe(III) changed systematically over time, and experimentally determined the strength of long-range temporal correlation in redox potential fluctuations at several stages of elapsed reaction time. Our results confirm that long-range correlation of redox potential fluctuations in biologically-influenced systems is stronger than long-range correlation in abiotic systems. Furthermore, we discovered that correlation strength dissipates as a logistic function of time and exhibits direct electrochemical dependence on free energies of reaction.

## Results

### Fe(II) oxidation kinetics

**Figure 1:**
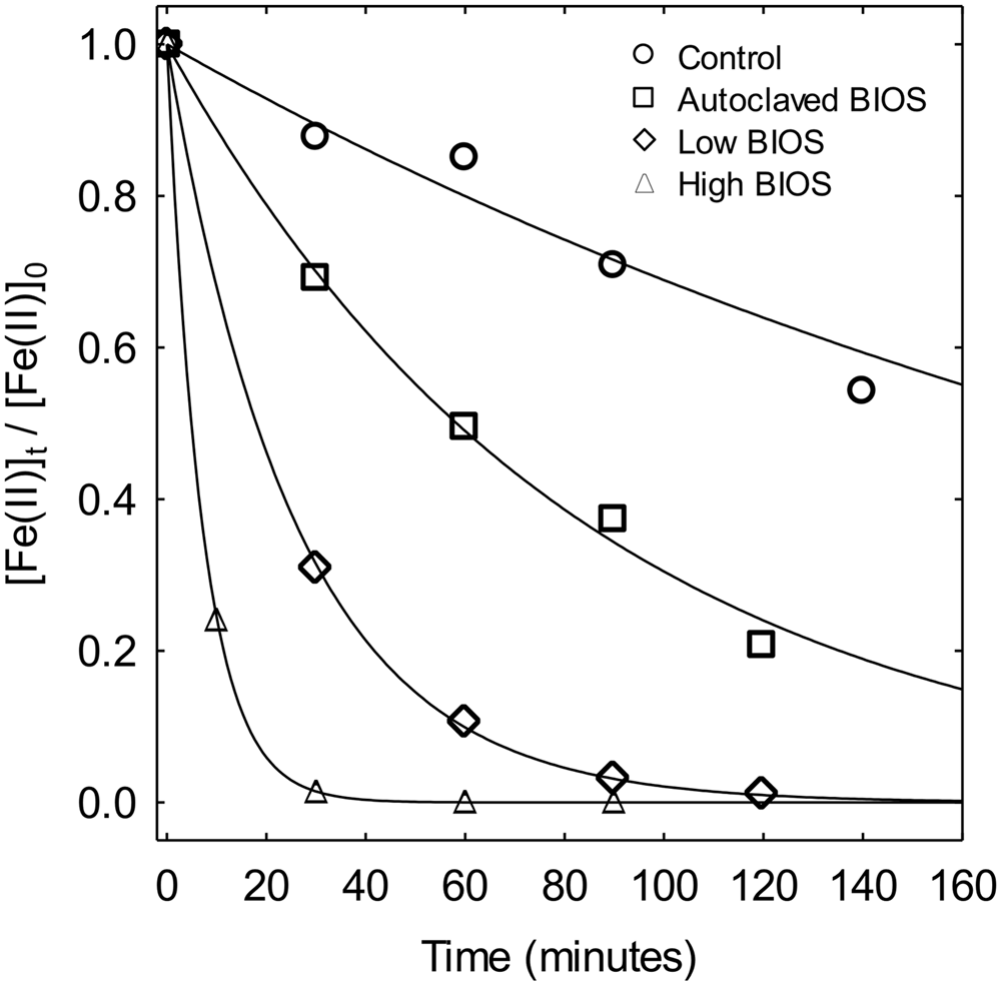
Decrease in relative Fe(II) concentrations as a function of time *t* in each of the four microcosms. The solid lines in the plot are best-fit curves for pseudo-first order kinetics of Fe(II) oxidation from non-linear estimation in STATISTICA v13.2; rate constant estimates are listed in Table 1.

The oxidation of Fe(II) in the microcosms followed pseudo-first order reaction kinetics, wherein relative Fe(II) concentrations decrease exponentially over time according to

1 $${[{\rm{Fe}}({\rm{II}})]}_{t}/{[{\rm{Fe}}({\rm{II}})]}_{0}=\exp (-{k}_{ox}t)\,$$

where *k*_*ox*_ is the pseudo-first order oxidation rate constant, and *t* represents elapsed reaction time (Fig. 1). The pseudo-first order rate constants obtained for Fe(II) oxidation in the microcosms are listed in Table 1, as are the corresponding mean recorded measurements of temperature, *p*H, and *p*O_2_ over the duration of the experiments. In each microcosm, *p*H and *p*O_2_ values exhibited little variation, as implicitly assumed for pseudo-first order Fe(II) oxidation kinetics. Estimates for *k*_*ox*_ ranged from 0.004 to 0.012 min^−1^ for the Control and Autoclaved microcosms and 0.039 to 0.141 min^−1^ for the Low BIOS and High BIOS microcosms, respectively. The temperature of the Autoclaved microcosm at 20.95 °C was slightly warmer than the others (ca. 16.00 °C), which is a result of cooling this sample to ambient temperature after autoclaving. Relative to the other microcosms, it is possible that the higher temperature of the Autoclaved microcosm contributed to a *k*_*ox*_ value higher by a factor of approximately 3, based on the Arrhenius relationship for the temperature dependence of reaction rates, and a reported activation energy of 134 kJ mol^−1^ for Fe(II) oxidation^24^.

**Table 1:** Microcosm pH, dissolved oxygen, and Fe(II) oxidation pseudo-first order rate constants.

Microcosm	Temp (°C)	*pH*	*pO*_2_ (atm)	*k*_*ox*_ (min^−1^)	*r* ^2^
Control	15.88 ± 0.72	6.40 ± 0.08	0.07 ± 0.02	0.004 ± 0.001	0.98
Autoclaved	20.95 ± 0.06	6.59 ± 0.01	0.09 ± 0.01	0.012 ± 0.001	0.99
Low BIOS	16.09 ± 0.21	6.39 ± 0.15	0.05 ± 0.04	0.039 ± 0.001	0.99
High BIOS	15.82 ± 0.94	6.20 ± 0.04	0.04 ± 0.01	0.141 ± 0.003	0.99

### Long-range correlations

**Figure 2:**
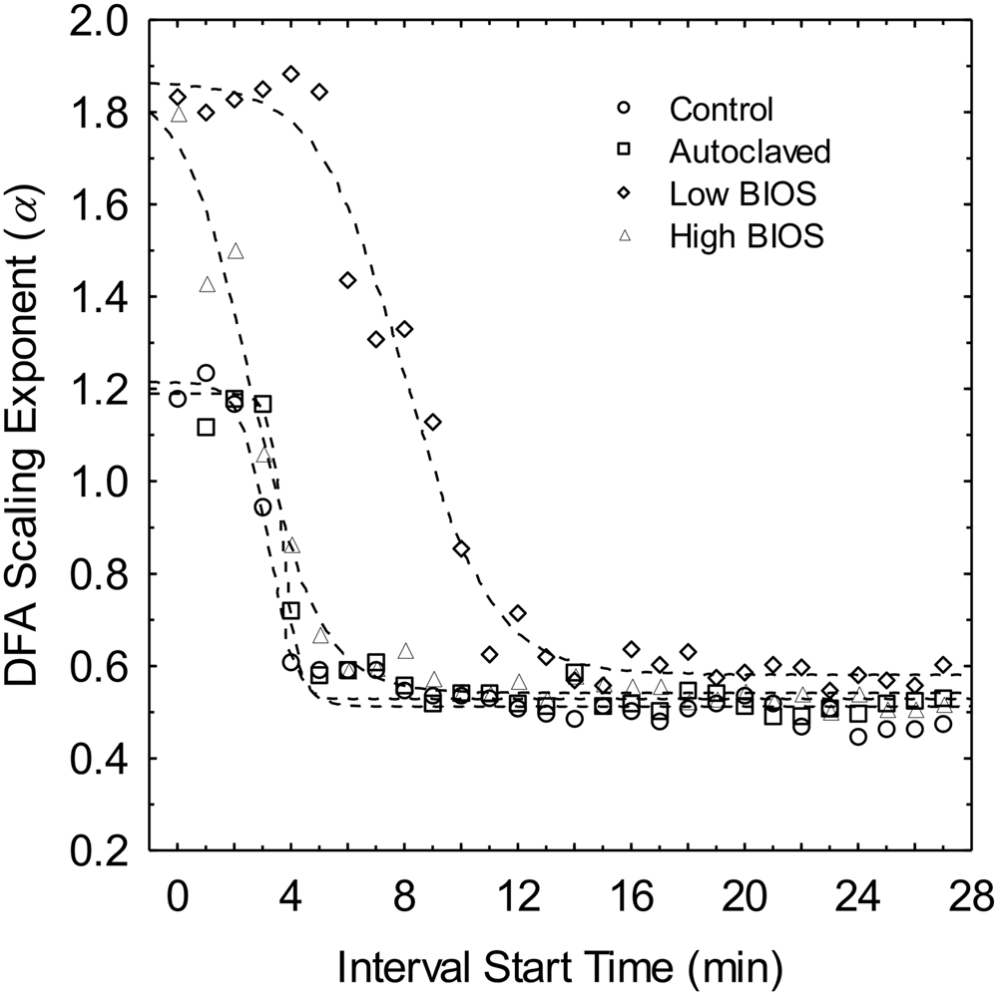
DFA scaling exponent (*α*) as a function of reaction time for each system. The dashed lines in the plot are best-fit curves for logistic decay from non-linear estimation in STATISTICA v13.2; parameter estimates are listed in Table 2.

The initial scaling exponents calculated for DFA start times of *t* = 0 in the Low and High BIOS microcosms (ca. 1.80) were evidently higher than those for the Control and Autoclaved BIOS microcosms (ca. 1.20) (Fig. 2). This is consistent with the expectation of stronger long-range correlations in redox fluctuations arising from microbial catalyzed Fe(II) oxidation compared to homogeneous or heterogeneous chemical oxidation in the Control and Autoclaved microcosms, respectively. While some variation also exists in initial scaling exponents between the Control and Autoclaved microcosms, the difference is not significant within the standard errors of the DFA *α* values (i.e., *p* > 0.05 in two-sided t-test).

As the oxidation of Fe(II) advanced with time, *α* values for all microcosms decayed sharply from high initial values to levels near 0.5 that typically signal random uncorrelated fluctuations in redox potential. The observed decreases *α* over time in the microcosms are characteristic of a generalized logistic decay function similar to the Fermi-Dirac distribution of particle energy states^28^

2 $${\alpha }_{t}={\alpha }_{min}+\frac{({\alpha }_{max}-{\alpha }_{min})}{1+\exp ({\beta }_{t}[t-{t}_{m}])}$$

where *α*_*max*_ and *α*_*min*_ represent maximum and minimum DFA scaling exponent values, respectively, *β*_*t*_ is the decay constant, and *t*_*m*_ is the position of the crossover inflection point of the curve where the maximum rate of decay occurs. Estimates for *α*_*max*_, *α*_*min*_, *β*_*t*_, and *t*_*m*_ are shown in Table 2. The *β*_*t*_ decay constants for the Control and Autoclaved microcosms were faster (ca. 2.00 min^−1^) than those determined for the Low and High BIOS microcosms (ca. 0.75 min^−1^), indicating that long-range correlations in redox potential may persist longer in bacterial mediated Fe(II) oxidation in comparison to homogenous and heterogenous abiotic reactions. Crossover threshold times were shortest for slow rates of Fe(II) oxidation in the Control and Autoclaved microcosms, as well as high rates of Fe(II) oxidation in the High BIOS microcosm. In remarkable contrast, the longest crossover threshold time was obtained in the Low BIOS microcosm with intermediate Fe(II) oxidation rates.

**Table 2:** Parameter estimates for the logistic decay of microcosm *α* values as a function of time.

Microcosm	*α*_*max*_	*α*_*min*_	*β*_*t*_ (min^−1^)	*t*_*m*_ (min)	*r* ^2^
Control	1.22 ± 0.03	0.51 ± 0.01	2.01 ± 0.39	3.22 ± 0.11	0.97
Autoclaved	1.15 ± 0.04	0.52 ± 0.01	2.02 ± 0.45	4.03 ± 1.15	0.97
Low BIOS	1.85 ± 0.04	0.58 ± 0.02	0.72 ± 0.08	8.28 ± 0.22	0.99
High BIOS	1.86 ± 0.11	0.54 ± 0.01	0.82 ± 0.12	2.61 ± 0.30	0.98

### Gibbs energy of Fe(II) oxidation

Gibbs energies for the oxidation of Fe(II) in the microcosms were calculated at elapsed reaction times corresponding to each *α* value as,

3 $${\rm{\Delta }}{G}_{r}={\rm{\Delta }}{G}^{0}+RT\,\mathrm{ln}(\frac{\{Fe(III)\}}{\{Fe(II)\}\{{H}^{+}\}p{O}_{2}^{0.25}})$$

with Δ*G*^0^ = −46.0 kJ mol^−1^ for the combined Fe(II)/Fe(III) and O_2_/H_2_0 half-cell reactions corrected from 25 °C to the measured temperature of the Control, Low BIOS, and High BIOS microcosms using the van’t Hoff equation^29^; at the temperature of the Autoclaved microcosm, Δ*G*^0^ = −45.1 kJ mol^−1^. Concentrations of Fe(II) were determined using Eq. 1 with corresponding oxidation rate constants and elapsed reaction times. The amounts of Fe(III) arising from the oxidation of Fe(II) over time were obtained from mass balance

4 $${[{\rm{Fe}}({\rm{III}})]}_{t}={[{\rm{Fe}}({\rm{II}})]}_{0}-{[{\rm{Fe}}({\rm{II}})]}_{t}$$

Concentrations were corrected to activities using the Davies equation^29^.

**Figure 3:**
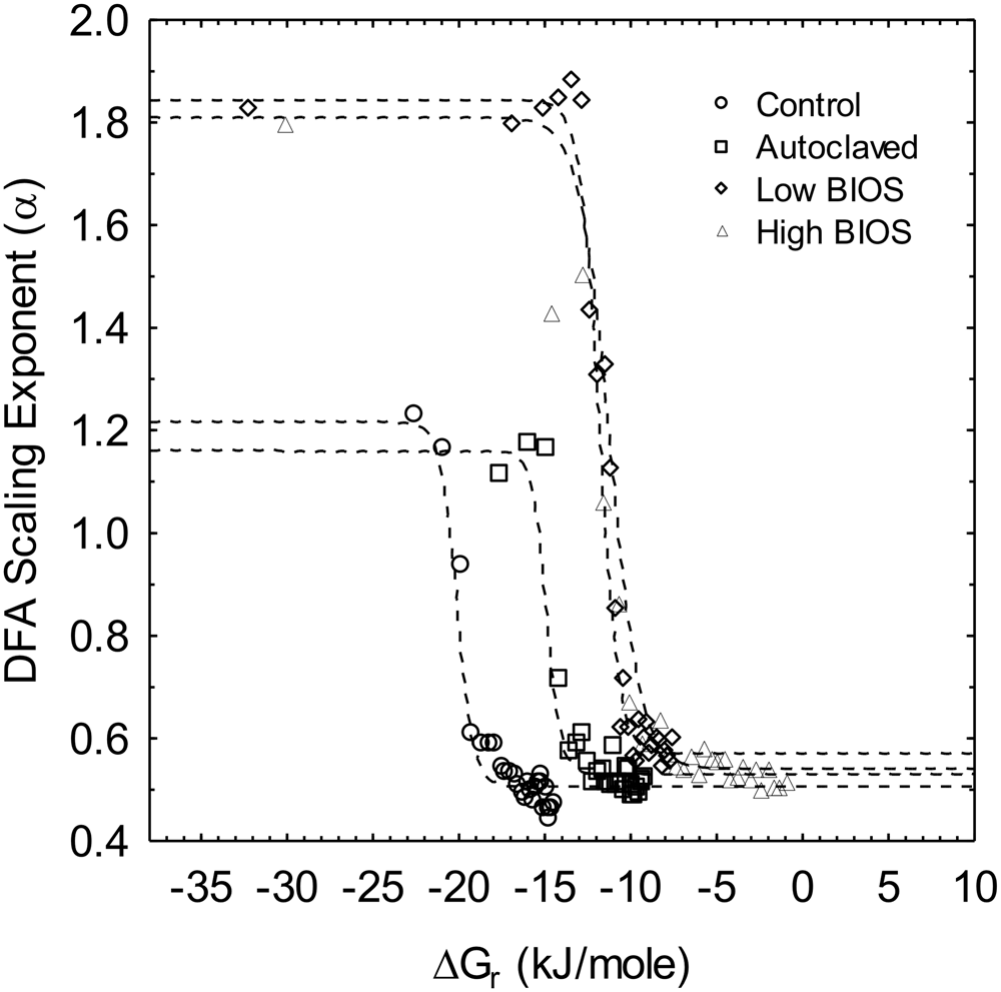
DFA scaling exponent (*α*) as a function of Δ*G*_*r*_ for each system. The dashed lines in the plot are best-fit curves for logistic decay from non-linear estimation in STATISTICA v13.2; parameter estimates are listed in Table 3.

Plots for the logistic decay of microcosm DFA scaling exponents in Eq. 2 using calculated Δ*G*_*r*_ values for Fe(II) oxidation as the independent variable instead of time are shown in Fig. 3; here Δ*G*_*m*_ is the change in free energy at the inflection midpoint. Unlike the time course results, this representation of the microcosm data displays a clear distinction between the controls and BIOS experiments. Overall, there is good agreement between *α*_*min*_ and *α*_*max*_ estimates obtained from nonlinear regressions of time course and free energy data in Eq. 2 (Tables 2 and 3). Also, the derived free energy *β*_Δ*Gr*_ decay constants for the Control and Autoclaved microcosms were slightly greater (ca. 2.25 mol kJ^−1^) than the Low and High BIOS microcosms (ca. 1.40 mol kJ^−1^). A more striking feature is the similarity in the Δ*G*_*m*_ crossover thresholds of −20.12 and −14.90 kJ mol^−1^ for the Control and Autoclaved microcosms, and particularly the Δ*G*_*m*_ values of −11.84 and −11.24 kJ mol^−1^ for the Low and High BIOS microcosms, respectively. The Δ*G*_*m*_ values are virtually the same as Δ*G*_*r*_ calculated on the basis of corresponding crossover threshold times (Table 3), which correspond to −19.90 and −14.23 kJ mol^−1^ for the Control and Autoclaved microcosms, and −11.47 and −12.09 kJ mol^−1^ for the Low and High BIOS microcosms. These observations emphasize not only the functional relationship between the temporal transition in long range correlation strength of Fe(II) oxidation redox potential fluctuations and reaction free energy, but also the clear distinction between chemical and biological Fe-based redox reactions.

**Table 3:** Parameter estimates for the logistic decay of microcosm *α* values as a function of Δ*G*_*r*_.

Microcosm	*α*_*max*_	*α*_*min*_	*β*_Δ*Gr*_ (mol kJ^−1^)	Δ*G*_*m*_ (kJ mol^−1^)	*r* ^2^
Control	1.22 ± 0.01	0.51 ± 0.01	2.10 ± 0.33	−20.12 ± 0.09	0.98
Autoclaved	1.16 ± 0.03	0.53 ± 0.01	2.37 ± 0.95	−14.90 ± 0.36	0.97
Low BIOS	1.84 ± 0.03	0.57 ± 0.02	1.79 ± 0.23	−11.84 ± 0.08	0.99
High BIOS	1.81 ± 0.03	0.54 ± 0.01	1.12 ± 0.07	−11.24 ± 0.08	0.99

## Discussion

The range of Fe(II) oxidation rate constant (*k*_*ox*_) estimates in this study (0.004 to 0.141 min^−1^) are in concordance with those determined from previous microcosm investigations, which span from 0.016 to 0.249 min^−1^ ^11,12,23^. Additionally, *k*_*ox*_ estimates of 0.141 min^−1^ for the High BIOS microcosm are about three times that of the Low BIOS microcosm (0.039 min^−1^; Table 1), as expected from other studies documenting the dependence of Fe(II) oxidation rates on BIOS concentration^11,22^. In tandem, the oxidation rate constants for the two BIOS microcosms are anywhere from three to thirty-five times higher than the Control (0.004 min^−1^) and Autoclaved (0.012 min^−1^) microcosms. These differences re-emphasize the ability of FeOB to outcompete the kinetics of abiotic homogeneous oxidation of Fe(II) in solution, as well as heterogenous Fe(II) oxidation on HFO precipitates^14,23^.

In keeping with previous work, the initial elevated DFA scaling exponents in the Low and High BIOS microcosms confirm that higher rates of bacterial catalyzed Fe(II) oxidation gives rise to stronger long-range correlations in redox potential fluctuations than slower abiotic reactions. As anticipated from these observations, the long-range correlations dissipated to a random uncorrelated state (i.e., *α* = 0.5) as rates of Fe(II) oxidation declined over time towards equilibrium in each of the closed batch microcosm. The logistic decay rate constants of scaling exponents for redox potential fluctuations were also more than twice as fast in the Control and Autoclaved microcosms than in their BIOS counterparts, demonstrating that long-range correlations in redox potential fluctuations tend to be more persistent for bacterial Fe(II) oxidation than abiotic reactions. This was particularly evident in the case of the Low BIOS microcosm, which exhibited the longest crossover threshold time of 8.28 min for the transition in from long-range correlation to random uncorrelated fluctuations in redox potential. On the other hand, the High BIOS microcosm displayed not only the shortest crossover threshold time, but also the highest rates of Fe(II) oxidation with consumption of Fe(II) and production of Fe(III) driving towards equilibrium faster than in the Low BIOS microcosm. These counterintuitive results are, in fact, consistent with the dependence of redox reactions on the mutual diffusion and collision frequency of reactants to form close-encounter complexes, with short separation distances more favorable to electron transfers^30^. If the redox reaction rate is high, as is expected under conditions far from equilibrium, then diffusion may become limiting. This situation is consistent with the initial stages of the microcosm experiments when fast Fe(II) oxidation rates yielded DFA scaling exponents symptomatic of long-range correlation in redox potential fluctuations (i.e., *α* > 0.5). Conversely, diffusion constraints are relieved by slower reaction rates, which diminished over time in the microcosms with a shift to random uncorrelated fluctuations in redox potential as Fe(II) concentrations decreased. Specifically, at the comparable crossover threshold times and sluggish rates of abiotic Fe(II) oxidation in the Control and Autoclaved microcosms, Fe(II) concentrations calculated from Eq. 1 were 0.98 and 0.95 times lower than initial values, respectively. For higher rates of bacterial Fe(II) oxidation in the Low and High BIOS microcosms, the same calculations show that initial Fe(II) concentrations decreased by equivalent factors of 0.72 and 0.69, respectively. These considerations not only add another level of distinction between bacterial and abiotic Fe(II) oxidation, but also offer reconciliation for the wide displacement in crossover threshold times witnessed in the Low and High BIOS microcosms^30^.

Transformation of the DFA scaling exponent results from the time domain to Gibbs energies of reaction revealed a hypothesized, but heretofore undocumented, characteristic of bacterial Fe(II) oxidation. The existence of long-range correlations in redox potential fluctuations under conditions far from electrochemical equilibrium (i.e., ${\rm{\Delta }}{G}_{r}\ll 0$) is evidently more pronounced for the involvement of bacteria than abiotic reactions. Additionally, strong long-range correlations in redox potential fluctuations arising from bacterial activity persist to much lower reaction free energy levels as equilibrium is approached (i.e., ${\rm{\Delta }}{G}_{r}\to 0$) than is witnessed in abiotic reactions. This is mostly likely explained by the well-documented efficiency of bioenergetic processes in which redox energy is conserved biochemically rather than being lost to the environment through thermal dissipation^31^.

Altogether these findings constitute a significant breakthrough in deciphering the complex dynamics of microbial Fe(II) oxidizing systems in natural systems through the fluctuation analysis-based differentiation of biotic and abiotic reaction pathways. While Fe(II) oxidation is an important redox transformation that is tied closely to the biogeochemical cycling of many key elements including C, N, O, and S^10,32–34^, microbial bioenergetic processes are characterized by an immense degree of diversity of energy sources (i.e., electron donors) and terminal electron acceptors in natural systems^1,35–38^. In this regard, the potential for using DFA to detect long-range correlation strength of redox potential fluctuations associated with bacterial metabolisms other than chemoautotrophic Fe(II) oxidation remains virtually unexplored and ripe for investigation. Recent estimates of the number of undiscovered microbial species living *in refugia* and ephemerally on Earth underscore the importance of developing new research methods which are not reliant on physical access and sampling^38^. The *in situ* nature of the electrochemical DFA method makes it a potentially revolutionary tool in furthering our understanding of microbial contributions to important biogeochemical processes such as Fe(II) oxidation that mediate the flow of energy and matter across our planet’s complex interconnected systems^39,40^.

## Methods

### Study Site

This investigation was conducted at Ogilvie Creek (46° 09′55.5″N, 77°37′23.8″W), approximately 10 km west of Deep River in the municipality of Laurentian Hills, Ontario, Canada. The creek is fed by surface outflow of a small lake, as well as discharge from an anoxic chalybeate groundwater spring that drains over a shallow 3.0 m reach into the main stream channel^23^. The discharge zone is characterized by a thick, rust-coloured flocculent BIOS mat, consisting of copious amounts of filamentous *Leptothrix ochracea* bacterial sheaths and less abundant helical *Gallionella ferruginea* stalks intermixed with Fe-oxyhydroxide mineral precipitates^13,17,23^.

### Microcosm Experiments

Samples of BIOS and water were collected separately in several sterile 60 mL syringes approximately 2.0 m downgradient from the spring source. Following the same protocol to replicate previous experiments on kinetics of bacterial Fe(II) oxidation^7,11,23^, a series of four individual 0.72 L batch microcosms were set up in 1.5 L plastic bottles as follows: (i) a chemical control consisting of 0.22 μm filtered creek water, measuring homogenous, abiotic Fe(II) oxidation (Control); (ii) a sterilized control with 310 mg/L of autoclaved BIOS (Autoclaved) and 540 mL of 0.22 μm filtered creek water, measuring autocatalytic Fe(II) oxidation; and two live microcosms, (iii) 330 mg L^−1^ (Low BIOS); and (iv) 1000 mg L^−1^ (High BIOS). The dry weight BIOS concentrations were determined at the end of each experiment by drying microcosm contents at 70 °C to a constant weight. Microcosms were filled gently, with syringes emptied slowly and below the liquid surface to minimize the exposure of the BIOS and water to the atmosphere.

Microcosm temperatures, pH, and dissolved O_2_ (DO) were recorded using a YSI 600 QS sonde. Dissolved Fe(II) concentrations were measured in triplicate samples taken at 10- to 30-minute intervals on 0.22 μm filtered aliquots using a HACH DR/890 colorimeter with HACH Ferrous Iron reagent. The standard errors of average Fe(II) concentrations for the triplicate samples were typically <±10.0%. To assess redox potential fluctuations, open-circuit voltage across a Pt | Ag-AgCl combination electrode was measured continuously at a sampling frequency of 10 Hz (i.e., 10 measurements per second) for the entire duration of each microcosm. The voltage measurements were recorded using a National Instruments data acquisition device (DAQ) controlled by a laptop running LabVIEW 6.0.

### Data Analysis and Modeling

Measured microcosm Fe(II) concentrations over time were normalized to starting (i.e., *t* = 0 Fe(II) concentrations to account for variations between the microcosms, then fit to Eq. 1 by nonlinear regression using Levenberg-Marquardt optimization in STATISTICA 13.2. This was to derive estimates and standard errors for pseudo-first order rate constants *k*_*ox*_. The same fitting routine was used to derive parameter estimates and standard errors for logistic decay of characteristic power law scaling exponents (i.e., *α* value) in Eq. 2 as functions of elapsed reaction time and Gibbs energy for Fe(II) oxidation.

DFA was implemented in MatLAB, as described previously^7,25^. The original 10 Hz time series for each DFA start time were down-sampled from 10 Hz to 1 Hz realizations and truncated to a standard window lengths of 1000 s before analysis. The set of scaling exponent estimates was subsequently averaged over all 10 realizations for the same standardized time interval. In order to compare changes in correlation strength with changes in oxidation rate over elapsed time in the microcosms, scaling exponents were calculated sequentially at one-minute intervals with index, *i*, for the first 27 minutes of elapsed time in each microcosm corresponding to DFA start times ${({t}_{i})}_{{\rm{i}}=0}^{27}$, ${t}_{i}={t}_{0}+i$.

## Data Availability

Data sets are available on EarthArXiv, 10.17605/OSF.IO/MV6HA.
